# Design and Validation of a Custom-Made Laboratory Hyperspectral Imaging System for Biomedical Applications Using a Broadband LED Light Source

**DOI:** 10.3390/s22166274

**Published:** 2022-08-20

**Authors:** Jošt Stergar, Rok Hren, Matija Milanič

**Affiliations:** 1 Jozef Stefan Institute, Jamova Cesta 39, SI-1000 Ljubljana, Slovenia; 2 Faculty of Mathematics and Physics, University of Ljubljana, Jadranska Ulica 19, SI-1000 Ljubljana, Slovenia

**Keywords:** biomedical optics, LED light-source, system calibration, hyperspectral imaging, spectroscopy, instrumentation

## Abstract

Hyperspectral imaging (HSI) is a promising optical modality that is already being used in numerous applications. Further expansion of the capabilities of HSI depends on the modularity and versatility of the systems, which would, *inter alia*, incorporate profilometry, fluorescence imaging, and Raman spectroscopy while following a rigorous calibration and verification protocols, thus offering new insights into the studied samples as well as verifiable, quantitative measurement results applicable to the development of quantitative metrics. Considering these objectives, we developed a custom-made laboratory HSI system geared toward biomedical applications. In this report, we describe the design, along with calibration, characterization, and verification protocols needed to establish such systems, with the overall goal of standardization. As an additional novelty, our HSI system uses a custom-built broadband LED-based light source for reflectance imaging, which is particularly important for biomedical applications due to the elimination of sample heating. Three examples illustrating the utility and advantages of the integrated system in biomedical applications are shown. Our attempt presents both the development of a custom-based laboratory HSI system with novel LED light source as well as a framework which may improve technological standards in HSI system design.

## 1. Introduction

Hyperspectral imaging (HSI) was originally implemented in remote sensing [1,2,3] and has found numerous applications in recent decades, especially due to the rapid development of acquisition techniques. Examples include vegetation type and water source detection [4,5,6], wood and wood product control [7], food safety and quality control [8,9,10,11], artwork authenticity and restoration [12,13], and, more recently, biomedicine [14,15,16,17,18,19,20,21]. HSI has a distinct advantage for biomedical applications, using non-ionizing radiation and capturing biochemical information sensitive to clinically relevant changes (e.g., angiogenesis, hypermetabolism) while also containing data on tissue structure encoded in scattering and polarization parameters [14,15,16].

Commercial systems are readily available for standard HSI and are highly useful in reflectance and transmittance imaging. However, these systems typically lack the modularity required for comprehensive HSI use, which would include profilometry, fluorescence imaging, and Raman spectroscopy [22]. With this in mind, we approached the design of a custom-made laboratory HSI system, which would be particularly geared toward biomedical applications, thus being equipped with a custom developed reflectance source that prevents sample heating and having adaptable spatial resolution and spectral resolution of a few nm.

The main objective of this study was twofold: (i) to develop and characterize a modular multi-modal HSI system incorporating a LED light source that enables imaging in a wide visible and near infrared spectral band ranging from 400 nm to 1000 nm, and (ii) to validate the custom-made laboratory HSI system, with the aspiration to pilot standardization of the calibration protocols, which would enable studies to be performed across different instruments. While the purpose of the article is to show technological advances in the development of HSI systems in general, we have additionally illustrated the usefulness of our HSI system by specific applications in the biomedical field.

While Gutiérrez-Gutiérrez et al. [23] have accurately pointed out that the development of custom-made HSI systems presents significant challenges, the systematic application of validation protocols provides a basis for future standardization that can also influence the development of commercial systems. For this reason, we considered the secondary goal of the study to make the system versatile enough to be attractive for wide range of applications.

In the Section 1 of the paper, we describe the design of the system and present a framework for the system calibration; in the Section 2, we discuss calibration and characterization results, and, finally, we show the verification of the system against a reference technique along with three examples of its use in biomedicine. Abbreviations used in the article are outlined.

## 2. Materials and Methods

This section introduces the framework of the protocol for calibration and verification of the HSI system. The development of a custom-made laboratory HSI system is presented in detail in Appendix A.

### 2.1. HSI System

In order to be used in biomedical applications, HSI systems need to fulfill the following requirements:

1. The system should be able to image samples of the size of a human hand with a field-of-view (FOV) of 20–30 cm and with the spatial resolution of approximately 100 µm. Additionally, it should be possible to vary both FOV and spatial resolution to suit a particular application.
2. The system should offer a spectral range from 400 nm to 1000 nm (dictated by tissue native chromophores, i.e., tissue components absorbing light), while the spectral resolution should be below 10 nm and preferably close to 1 nm in order to study the fine spectral features of chromophores.
3. The illumination system should not heat samples, while the object’s shape and thickness information should also be obtained to enable quantitative imaging in transmission and reflection geometry using corrections for sample curvature and thickness.

**Figure 1:**
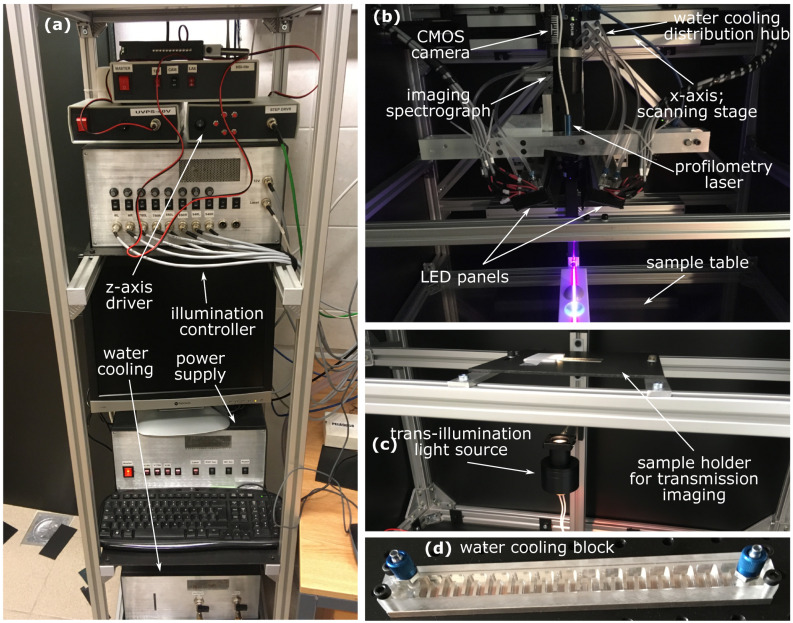
The custom-made laboratory hyperspectral imaging (HSI) system with annotated components: (**a**) drivers, power supply and water cooling; (**b**) reflectance imaging system illuminating two phantoms with a profilometry laser; (**c**) setup for transmittance imaging; (**d**) a single LED, water-cooled, broadband LED illumination module from the cooling block side [24,25,26,27,28,29].

To accommodate samples of different sizes, we propose standard push-broom imaging methodology, in which the scanning direction could be freely adjusted. This methodology enables acquisition of all the spectral channels along a spatial line simultaneously by means of an imaging spectrograph but requires scanning to obtain a full spatial image. The details of our custom-made laboratory HSI system (Figure 1) are given in Appendix A.

### 2.2. Verification and Calibration Protocols

An important aim of the research presented in this paper is to propose calibration and verification protocols that would, ultimately, enable quantitative measurements and correspondence between different systems at different settings; we need to acknowledge that much work has already been accomplished in this regard by studies proposing precise methods for calibration [30,31]. Specifically, we attempt to provide a protocol appropriate for biomedical applications, which is easy to understand and implement while still offering a sufficient degree of calibration and characterization rigor without compromising imaging quality.

In calibrating and characterizing a spectral imaging system, two separate stages need to be considered. First, spectral calibration must be performed, which includes calibration of the spectral axis to reference sources as well as determining the actual spectral resolution dictated by the quality of both the camera and optical components. Second, the spatial calibration, which relates detector pixel coordinates to distances in nature, as well as determination of actual spatial resolution must be performed. Again, the two can, and in fact will, diverge due to the quality of the optical components and their characteristics, such as imaging spectrograph slit size.

When performing real-life measurements, one must also consider the nature of the samples being imaged. Samples are usually extensive and must be illuminated with an appropriate light source. For the best quality of the results obtained from a system, one must consider effects such as the homogeneity of illumination and light source stability; light sources generate heat during the use and, thus, their spectral characteristics can change, which is the most unwelcome event. Additionally, samples can have a surface that varies in the source-detector separation, which can cause artifacts due to different heights and curvatures [27]. Additionally, this means that spectrograph and illumination properties must be tested at different distances between the object and the imaging lens.

Both spectrograph and the objective lens can introduce artifacts into the recorded data, such as vignetting (darkening of the peripheral regions of the image), spectral smile (bending of the spectral axis) and keystone (different magnifications at different wavelengths) [32]. These effects should be tested and evaluated. Small vignetting and illumination inhomogeneity can be eliminated in the data processing by normalization to a white reference (e.g., using a sample with almost 100% reflectance), but should be minimized nonetheless because a smaller amount of light collected from a region of the sample inherently reduces the signal-to-noise ratio and, thus, quality of the data.

Finally, the performance of a system should be checked using well-known samples by comparing the results to an established and trusted method. In this way, the performance of the system is verified.

**Figure 2:**
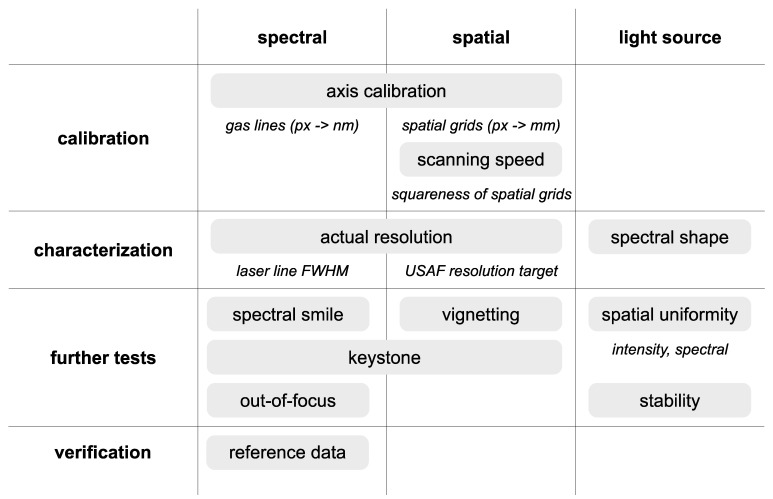
Outline of calibration, characterization, verification and further testing. In the calibration step, the spatial and spectral axes are calibrated using references; for the push-broom system, scanning speed is also calibrated. As part of the characterization step, actual spectral and spatial resolutions are determined, along with the spectral shape of the light source. Further steps include testing for spectral smile (bending of the spectral axis), keystone (different magnifications at different parts of the spectral axis), out-of-focus behavior and vignetting (darkening of the peripheral regions); additionally, light sources should be tested for spatial uniformity, both in terms of their intensity and spectral properties as well as temporal stability. In the verification step, the system is verified against known reference data, such as ink samples with known absorption properties.

Considering these aspects, we propose a set of tests that can also serve as a checklist for calibration, characterization and verification of HSI systems in general (Figure 2). The steps, denoted in gray boxes, should be taken in sequence from top to bottom and from left to right. In this way, any HSI system needs to be first calibrated, then characterized, checked for aberrations and spectral or spatial artefacts and, finally, verified.

## 3. Results

### 3.1. Spectral Calibration and Characterization

**Figure 3:**
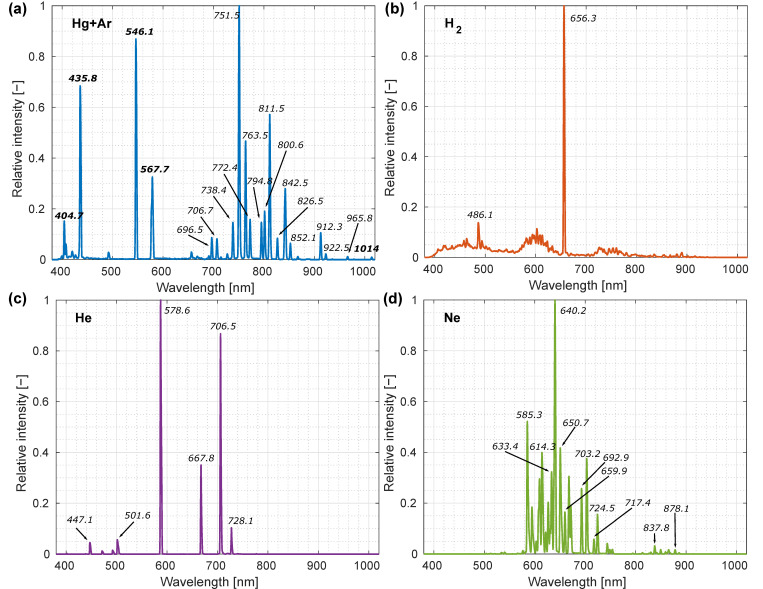
Gas vapor emission spectra with characteristic spectral lines used for the system spectral calibration are displayed for four different gases: (**a**) mercury vapor (with argon as an inert gas), (**b**) hydrogen, (**c**) helium and (**d**) neon. Spectra are acquired using the custom-made laboratory hyperspectral imaging (HSI) system; the calibrated wavelength *x*-axis is used to plot the data; lines that were used for calibration are annotated with characteristic wavelengths in air.

Since the geometry of the spectrograph optics was not precisely known, the relation between the dimensionless pixel index on the detector *x* and the wavelength of the incoming light *λ* was approximated using a third order expansion in the form of

(1) $$\lambda \left(x\right)=a+bx+cx^{2}+dx^{3}$$

where parameters *a*, *b*, *c* and *d* had to be determined through calibration. Parameters in Equation (1) were calculated using spectral sources with known spectral shapes. Light sources for the calibration were spectral tubes filled with helium, hydrogen, neon and mercury vapor (with argon as an inert gas) (Frederiksen Scientific, Olgod, Denmark), powered by a spectral tube holder and power supply (285550, Frederiksen Scientific, Olgod, Denmark); twenty hyperspectral images of each spectral tube were acquired, and results were averaged. Recorded spectra with marked characteristic spectral lines used for calibration are shown in Figure 3; data acquired were compared to the NIST database of characteristic gas emission line wavelengths [33].

**Figure 4:**
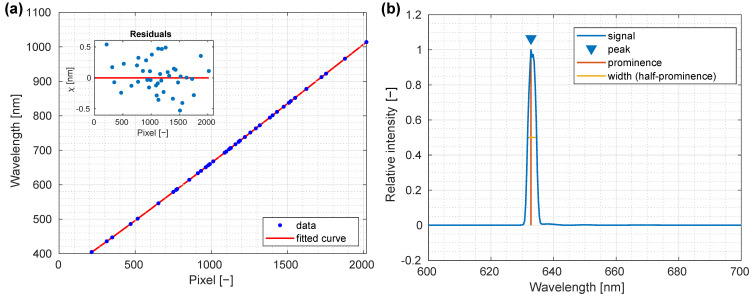
Spectral characterization of a custom-made laboratory hyperspectral imaging (HSI) system. (**a**) Fitting of 40 different gas spectral lines to determine the corresponding detector pixel index. (**b**) An averaged spectrum of a He–Ne laser along with the determined full width at half maximum (FWHM) (2.9 nm). Note the asymmetry of the laser line, which is due to the broadening of the peak.

The calibration process itself was performed iteratively, since the lines in some gases (argon and neon most notably) could be separated only by a few nm and could not be identified without an estimated central wavelength. The first calibration was performed by identifying spectral lines in the mercury and hydrogen spectra, whose tabulated line wavelengths [33] were used to determine parameters in Equation (1) and to calibrate the scale. In the next step, forty lines from all the spectra were identified based on their apparent intensity and estimated central wavelengths; these data were then used to perform a precise determination of the parameters in Equation (1), with the following values obtained: *a* = 337.7 ± 0.8 nm, *b* = 0.306 ± 0.003 nm, *c* = (2.6 ± 0.3) 10^−5^ nm and *d* = (−5.4 ± 0.8) 10^−9^ nm. As can be seen in Figure 4a, depicting the fit of Equation (1) to the data, the residuals were randomly distributed around zero with maximal deviations of 0.5 nm (2 pixels on the detector), which could be attributed to the limited resolution of the spectrograph or possible error when determining the center of lines in the recorded spectra.

The manufacturer specified a value of 2.5 nm as the spectral resolution of the spectrograph. Testing the calibration of the system as well as evaluating the spectral broadening in the recorded spectra, a helium–neon laser (HNL050L, Thorlabs, Newton, NJ, USA) was directed into a block of Teflon (PTFE, Dastaflon, Medvode, Slovenija), and the scattered light was recorded using the spectrograph. Recording the scattered light increased the size of the beam while the light intensity was decreased, preventing saturation. Measured spectra were averaged over the central area of the resulting illuminated spot over 20 images (Figure 4b), and from this average spectrum, the laser line wavelength and the line width were determined; the peak intensity was at 632.8 nm, which was in a perfect agreement with the laser specifications. It is important to note, however, that the peak was broadened across approximately three pixels, thus, the detected wavelength uncertainty was approximately 1 nm, estimated from fitted parameters and Equation (1). The resulting spectral width was 2.9 nm, which was larger than specified by the manufacturer, however, the error in determining the width of the line could be up to two pixels, which corresponded to 0.6 nm, thereby, the measured width agreed with the specified spectral resolution provided by the manufacturer within the precision.

### 3.2. Spatial Calibration and Characterization

To calibrate the spatial dimension of the system, a set of calibration standards (Ronchi grids and a USAF1951 target) was created in graphics software (Inkscape) and printed in a 1:1 ratio on a laser printer. The dimensions were verified using multiple calipers and rulers after printing. Calibration patterns consisted of a set of different Ronchi grids with alternating bright and dark lines with spatial frequencies of 2 lp/mm, 1 lp/mm, 0.5 lp/mm and 0.2 lp/mm. Spatial frequency was defined as 1/*d* = 1/2 *w* (lp/mm), where *d* was the distance between two edges of two different lines and *w* was the width of the line in millimeters; widths were the same for dark and bright lines.

All images for the spatial calibration were acquired by the full LED illumination, and the spectral band at 500 nm was used for analysis.

First, Ronchi grids with 1.0 lp/mm and 0.5 lp/mm were imaged and distances in pixels were measured and compared to the real distances given by spatial frequencies. These values were then used to calibrate the length scales of the instrument (Table 1). Each measurement was repeated twice, once for each line spacing. These measurements showed that for the 17 mm lens, one pixel on the detector corresponded to 0.12 mm on the object plane, whereas for the 50 mm lens, one pixel on the detector corresponded to 0.03 mm on the object plane.

**Figure 5:**
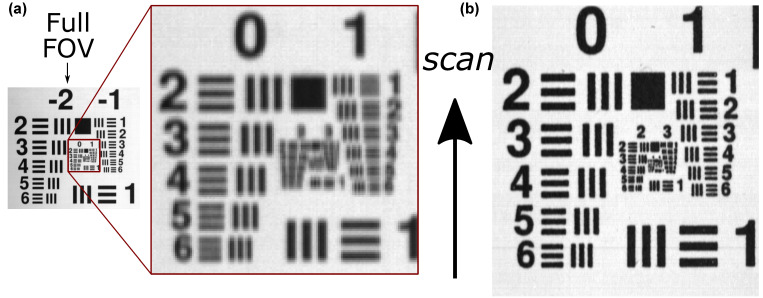
Resolving power test for custom-made laboratory hyperspectral imaging (HSI) system employing a USAF1951 resolution chart. (**a**) The image recorded by a 17 mm lens with magnified region that corresponds to FOV of a 50 mm objective lens; (**b**) the same image recorded by a 50 mm lens. Scanning direction is marked with an arrow; the hyperspectral images were taken by the full LED illumination; the presented images correspond to the spectral band at 500 nm.

The system resolving power (minimal spatial frequencies discernible using the instrument) was evaluated by means of a USAF1951 target, printed with groups of −2 to 3 visible (Figure 5). For clarity, the same group that was visible with a 50 mm lens was cropped and magnified for a 17 mm lens. The smallest discernible element for a 17 mm lens at the 500 nm band was the group 0 element 5, which corresponded to a spatial frequency of 1.59 lp/mm and discernible features with characteristic size of about 0.3 (1 ± 0.1) mm. Within the precision of the USAF1951 resolution target test, the same resolving power was observed at 850 nm. For a 50 mm lens, the group 2 element 3 was still discernible in both directions at 500 nm, which corresponded to a spatial frequency of 5.04 lp/mm and the smallest observable features in the range of 0.1(1 ± 0.1) mm. At 850 nm, a decrease in resolving power was observed, with group 1 element 1 still discernible, amounting to a spatial frequency of 2.0 lp/mm and discernible features of 0.25 (1 ± 0.1) mm. It is noteworthy that the resolving powers in both directions of the image were not the same; along the scanning direction, resolution was limited by the spectrograph entrance slit, whereas along the scan-perpendicular direction, the limiting factor was the detector pixel size. Thus, resolution perpendicular to the scanning direction was better than that along the scanning direction, which can be ascertained in Figure 5 as a clear separation of vertical lines, whereas horizontal lines are already blurred. For the overall system resolution, we have conservatively taken the resolution in the scanning direction.

**Figure 6:**
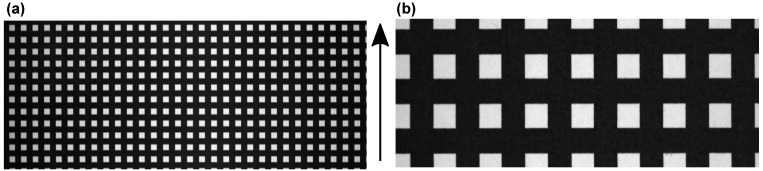
Scanning speed calibration. Images of a 5 mm grid are recorded with (**a**) a 17 mm lens and with (**b**) a 50 mm lens. Direction of the scan is shown with an arrow.

The scanning speed, in combination with the camera exposure time, sets the aspect ratio of scan-parallel and scan-perpendicular axes. To achieve the same distance calibration in both axes, the scanning speed has to be adjusted precisely in accordance with the exposure time of the camera. For scanning speed verification, images of a square calibration grid with spatial frequency of 0.2 lp/mm were recorded for both 17 mm and 50 mm lenses, and the scanning speed was adjusted to obtain the image pixels as close to the square shape as possible (Figure 6). Ideally, sizes in both dimensions should be the same, however, as the speed of the stage could only be adjusted in discrete steps, it was not possible to achieve this completely. After the calibration, elongation along the scan direction of about 6% for a 50 mm lens was measured, whereas for a 17 mm lens, elongation was about 1 pixel, which was approximately the precision of the system.

### 3.3. Spectral Smile and Keystone Analysis

**Figure 7:**
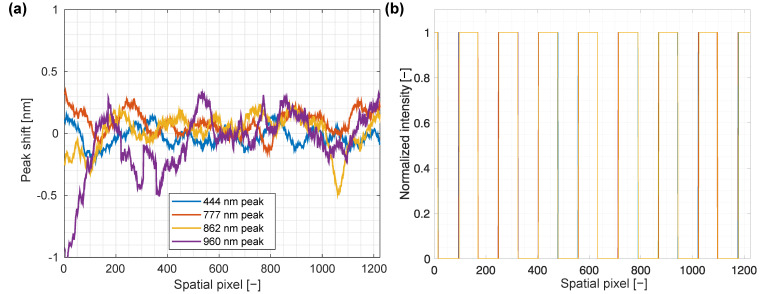
(**a**) To determine spectral smile, pixel coordinates of maxima in the spectra corresponding to peaks of different LEDs comprising the illumination were tracked at different spatial coordinates, and the offset from the wavelength at the center of the FOV was calculated. To improve signal-to-noise ratio, a running average of 50 elements was applied to the calculated shifts. (**b**) An example of keystone deformation determination for a 50 mm objective lens shows a view across the spatial axis of an HSI of a square grid pattern. Cross-sections at different wavelengths are plotted on the same graph with different colors, showing an almost perfect agreement.

When using an imaging spectrograph, a characteristic aberration called the spectral smile can be present; it is the bending of the spectral axis along the spatial axis that is observed as a different spectral shift in different spatial bands. To test for the spectral smile, a large, 4 cm thick PTFE slab (Dastaflon, Slovenia) was illuminated by the LED light source. The characteristic peaks were first identified in the spectra for each spatial pixel; next, the mean value of the central wavelength in the middle of the spatial axis was subtracted and a running average of 50 elements was applied to the resulting offset to make the analysis easier. Finally, the resulting smoothed offsets of the central wavelengths were plotted against the spatial pixel coordinate, as shown in Figure 7a. No significant bending of the spectral axis was present; thus, the spectral smile was smaller than the pixel size of 3.45 µm, which agreed well with spectral smile below 1.5 µm specified by the manufacturer. Deviations from the line could be attributed to both the system resolution of around 0.3 nm and possible differences between LEDs that occurred during the manufacturing process.

Since spectral keystone can cause mixing of spectra from neighboring pixels, it was evaluated on a single hyperspectral frame (one spatial and one spectral dimension) across the white squares in Figure 6. The edges of the squares, as shown in Figure 7b, were inspected for shift across the whole spectral range, and shifts in positions corresponding to keystone were measured. For a 50 mm lens, keystone was below 2 px over the whole range, thus falling within the measurement precision. For the 17 mm lens, the central half of the image exhibited keystone below 2 px, whereas at the edges, 5 px deformation was observed. This small deformation did not present an obstacle for instrument use since it was comparable to actual instrument resolution.

### 3.4. Testing Lens and Out-of-Focus Effects

**Figure 8:**
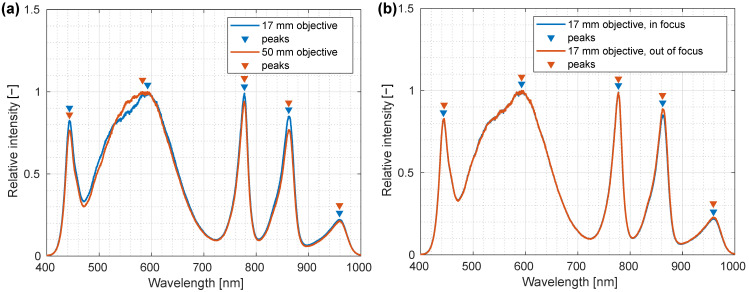
Verification of in-focus and out-of-focus spectral calibration of a custom-made laboratory hyperspectral imaging (HSI) system. (**a**) LED illumination spectra for 50 mm (red line) and 17 mm (blue line) objective lenses. Some differences in spectral shape that are due to different objective lens coatings are also visible in the recorded spectra. Most importantly, a 17 mm objective lens had a slightly decreased transmittance between the 550 nm and 600 nm when compared to a 50 mm objective lens. (**b**) Spectra recorded at a focus plane and 10 cm above the sample recorded using a 17 mm objective lens. Detected peak intensity wavelengths are annotated on the plots and are mostly independent of the distance between the light source and detector.

Since either 17 mm or 50 mm objective lenses could be used, depending on the desired resolution and FOV, spectral calibration was tested for both lenses. To evaluate possible differences in spectral calibration when exchanging lenses, peaks in LED illumination were used. A calibrated white standard (PELA 9058, PerkinElmer, Waltham, MA, USA) was illuminated with the integrated LED light source, and spectra at the center of the standard were measured with both lenses. Twenty raw spectral images (one spatial and one spectral dimension) were acquired from the area of the white standard and averaged to decrease the effects of random noise, while local maxima were identified using MATLAB (Figure 8). Good agreement between both lenses was observed with minimal deviations within the instrument precision. For the white LED, the change in the peak shape due to the objective transmittance caused a disagreement, as seen in Figure 8a.

The effects of focus distance were tested using a 17 mm objective lens. Spectra were recorded following the same protocol as when testing lens effects for an in-focus position and for a position 10 cm above the focus. Peaks were identified and central wavelengths were calculated using MATLAB. Good agreement was observed, indicating minimal changes in the illumination spectral profile with distance between the light source panels, object imaged and the detector, as demonstrated by Figure 8b.

### 3.5. Spatial Homogeneity of Illumination

The custom-made LED light source should illuminate the sample homogeneously in terms of both spectral shape and intensity. Spectral homogeneity was tested indirectly when examining the spectrograph for spectral smile. While small differences in the spectra, seen as shifts of the peak wavelength, were observed, they were mostly below 0.5 nm, and thereby close to the system resolution.

**Figure 9:**
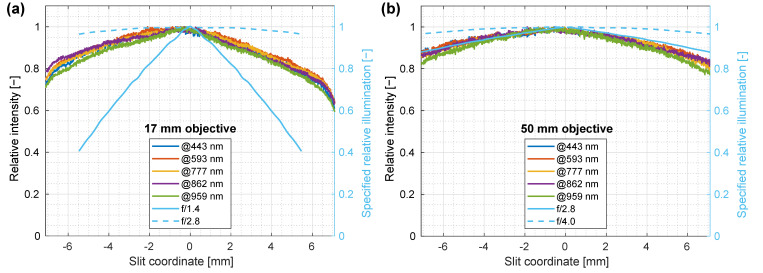
Spatial homogeneity of LED illumination for (**a**) 17 mm and (**b**) 50 mm objective lenses. Recorded normalized intensity is compared to the objective lens transmission specified by the manufacturer (blue color). To reduce the noise, an averaging of 30 measurements for each lens was performed.

Spatial intensity homogeneity is the measure of deviation from the average illumination intensity value across the imaged FOV and an indication of the presence of the local illumination variations; the first is called the non-equal illumination and the second corresponds to brighter areas due to the LED illumination geometry. Intensity homogeneity was tested by measuring the reflected light from a 3 cm thick slab of PTFE for both objective lenses, while averaging 30 measurements for each lens. The PTFE slab was deliberately chosen for this test since the white standard (PELA 9058, PerkinElmer, USA) was smaller than the FOV of the system with a 17 mm objective lens, and it would be thus impossible to cover the whole FOV in one measurement; the slab was also sufficient for assessing spatial homogeneity despite not having 100% reflectivity in the whole spectral range. Recorded normalized intensity distributions were then compared to the transmission values of the objective optics (detector relative illumination—the amount of light that a lens collects on a specific part of the detector, combining the effects of vignetting and roll-off, given a homogeneous imaging FOV) specified by the manufacturer (Figure 9).

The aperture for a 17 mm lens was set between 1.4 and 2.8 f-stop gradation, which explains the decline in intensity from the center in Figure 9; the rapid drop in the detected light at the edges of the field could be attributed to vignetting. For a 50 mm lens, the aperture was set at approximately f/2.8, which was the lowest f-stop value for this objective lens. In this case, the measured intensity on the detector agreed almost perfectly with the specified relative illumination provided by the objective lens manufacturer.

The skew of the spatial profiles could be attributed to a slightly inhomogeneous illumination (at 10% difference), which was compensated for when processing real data by means of normalization with a white reference spectrum. Additionally, both distributions in Figure 9 showed an apparent shift in slit coordinate position between maximum detected intensity and maximum relative illumination, as specified by the manufacturer of the objective lens. This asymmetry could be attributed to an imperfect centering of the objective lens on the spectrograph optical axis. This, however, did not influence the recorded image quality, since these slight variations were mitigated by normalization of recorded spectra to a white reference.

### 3.6. Illumination Composition and Temporal Spectral Stability of LED Light Source

**Figure 10:**
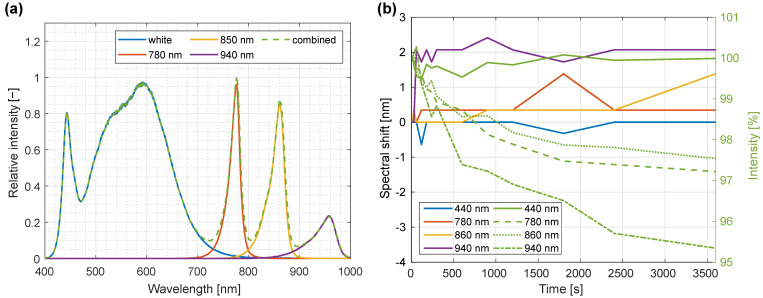
Illumination composition and temporal spectral stability of LED light source. (**a**) Different individual LED spectra along with the complete illumination spectrum. (**b**) Temporal stability in terms of the spectral shift and intensity change at different time intervals compared to the state 10 s after turning the illumination on. To reduce the noise, averaging of 30 measurements for each LED was performed.

The spectral composition of the LED illumination was evaluated by recording hyperspectral images of a white reference standard for each illumination LED type and the whole LED panel. Figure 10a shows a plot of illumination spectra for individual LEDs as well as for complete illumination.

For verification of spectral temporal stability of the LED light source, white reference images of PELA 9058 standard were recorded at different time intervals after turning the illumination on. Spectra were normalized to the dynamic range of the detector, with peak positions and normalized intensity values detected using *findpeaks* MATLAB function. From these data, spectral shifts were calculated by subtracting from the first measurement the values obtained at later time intervals; similarly, for intensity, data recorded at later times were divided by the value at the start (Figure 10b).

During the first minute after the light source was turned on, a rapid change was observed in both central wavelengths and intensity. During the time interval between 1 min and 10 min, central wavelengths consistently changed by less than 0.5 nm, which indicated the validity of a recorded white reference for at least 10 min. The intensity at the peak decreased approximately exponentially with time for the near infrared (NIR) LEDs, with an estimated warm-up time of 30 min. After that, changes became gradual enough (relative intensity change of less than 1% during a 10-min period) and did not disturb the measurements. For the visible LEDs, this trend was even less pronounced, with relative changes of intensity below 1% for the whole 10-min period.

### 3.7. Verification against a Reference Instrument

The system was verified by imaging a set of liquid dye samples sandwiched between two microscopy glasses. Samples were also measured using a reference laboratory spectrometer that served as the gold standard. The sample cells were prepared in a standard fashion for microscopy of liquid samples as used in microrheology and microfluidics [34], which is outlined in more detail in Appendix B.

For the reference measurement of sample cells, a PerkinElmer Lambda 1050 UV/VIS/NIR spectrometer (PerkinElmer, Waltham, MA, USA) was used with a PerkinElmer spectrometer add-on 3D WB detector module (PerkinElmer, Waltham, MA, USA) specifically installed for optical transmission or absorption measurements of liquid and solid samples in the range of 175–3300 nm. For verification, blue and red ink were employed, since their respective spectra were not overlapping. The sample cells were placed against the cuvette holder in the spectrometer and affixed to it using a piece of masking tape to prevent movement during the measurement. A spectral range between 400 nm and 1050 nm with a step size of 1 nm was selected. The detector switch between the InGaS sensor and Si photodiode occurred at 860 nm. Recorded spectra were corrected for the detector switch, the values were kept in the visible range and an offset to the reflectance was added in the NIR region to obtain a smooth curve. Spectra were measured in the collimated transmittance mode and normalized to an unobstructed beam. To reduce the signal noise, a 10-signal averaging was turned on.

**Figure 11:**
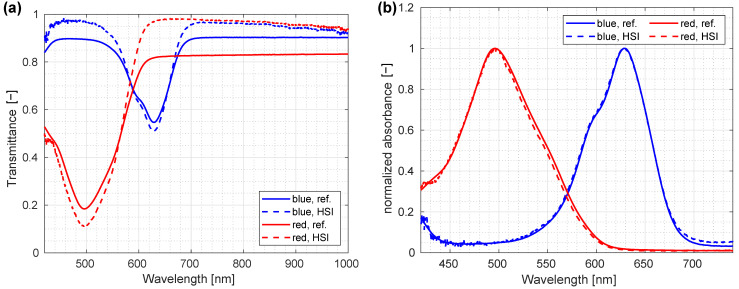
Comparison between the transmission spectrum of a custom-made laboratory hyperspectral imaging (HSI) system and reference spectrum: (**a**) direct comparison of spectra; (**b**) comparison of normalized absorbance.

The sample cells were imaged using the transmission modality of the custom-made laboratory HSI system, with spectra for red and blue ink measured from the central part of the sample and averaged; for normalization, an image of the diffusive opaque plexiglass was employed. In this way, performance of the spectrograph was validated with geometry closely mimicking the reference system. A comparison between the transmission spectrum of HSI system and the reference system is shown in Figure 11a. All presented spectra were calculated as an average of a 20 × 20-pixel image area obtained from a spatially homogenous image region.

Although all previous measurements were performed in the reflectance mode, the verification against a reference instrument was performed in the transmittance mode. One of the reasons is that the liquid dye samples used in the verification were more appropriate for transmission imaging. At the same time, the reference spectrometer acquired images in transmission mode, thus, the differences in the spectral shapes due to different acquisition geometries were smaller if both measurements were performed in the same mode. This did not substantiate the system’s precision and accuracy in comparable terms for reflectance imaging directly, but the key performance indicator was the spectral performance of the spectrograph itself, not the system as a whole.

Since we expected that direct agreement between the HSI and reference spectra would be inadequate, mostly due to non-homogeneity of sample cells, normalized absorbances were compared instead of absolute transmittance values. Normalized absorbance was obtained by taking the logarithm of the transmittance value *T* and normalizing it to the maximal value,

(2) $$\tilde{A}\left(\lambda \right)=\frac{\ln\left(T\left(\lambda \right)a\right)}{\max\left[\ln\left(T\left(\lambda \right)a\right)\right]}$$

where *a* was the normalization factor accounting for different modalities, which were collecting different amounts of light. The normalized absorbance approach eliminated effects of differences in thickness and concentration of the samples and had proven useful to verify the shape of the features and their central wavelengths (Figure 11b).

The normalization factor, determined by fitting, was 1.19 for red ink and 1.09 for blue ink. The resulting normalized absorbance showed excellent agreement between the normalized absorbance obtained by custom-made HSI system and reference spectrometer (Figure 11b).

### 3.8. Example #1 of HSI-System Application in the Biomedical Field: Imaging of a Human Hand

**Figure 12:**
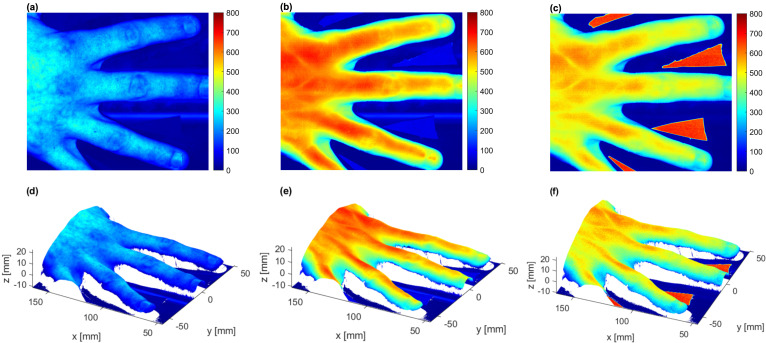
An example of utility of the custom-made laboratory hyperspectral imaging (HSI) system, where reflectance imaging and profilometry were simultaneously applied to a human hand. Raw hyperspectral images of a human hand at (**a**) 530 nm, (**b**) 770 nm, and (**c**) 930 nm with corresponding three-dimensional surface data: (**d**) 530 nm, (**e**) 770 nm, and (**f**) 930 nm are presented. The units are digital numbers. Profilometry measurements were aligned to the corresponding hyperspectral image. Information on hand thickness was collected at the pixel level. Integration time was 100 ms, FOV was 153 mm × 131 mm, with the entire imaging taking 210 s to acquire. The resolution of the hyperspectral image was 2448 × 2100, and resolution of profilometry was 0.17 mm in the scanning direction and 0.35 mm in the perpendicular direction.

To demonstrate the utility of our custom-made laboratory HSI system, we imaged a human hand using simultaneous reflectance imaging and profilometry. The profilometry measurements were aligned to corresponding hyperspectral images. Figure 12 shows hyperspectral images and corresponding three-dimensional surface data acquired at 530 nm, 770 nm and 930 nm; technical specifications are provided in the caption of the figure. One can clearly discern blood vessels and locations of joint gaps, skin folds and hand shape in general from the images, demonstrating both spatial and spectral sensitivity of the system that can be used for biological applications such as detection and monitoring of arthritis in small joints [35].

### 3.9. Example #2 of HSI-System Application in the Biomedical Field: Murine Tumor Model

In the second example, we used the murine tumor model. A BALB/c (BALB/cAnNCrl, Charles Rivers) 8–10 weeks old female mouse was implanted with a CT26 murine colon carcinoma (ATCC) to monitor the growth of a subcutaneous tumor. The experiment was approved by the Ministry of Agriculture, Forestry and Food of the Republic of Slovenia (permission no. U34401-36/2020/7). Detailed information on the mouse model can be found in [36].

**Figure 13:**
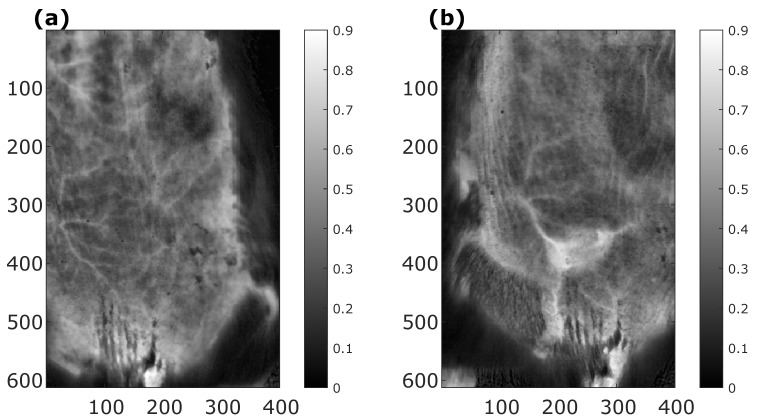
An example of utility of the custom-made laboratory hyperspectral imaging (HSI) system, with shown erythema index maps of a murine tumor model (**a**) before the tumor cells’ implantation and (**b**) six days after the implantation. Erythema index maps were calculated from recorded hyperspectral images. Since the erythema index is a ratio, it does not have units. Blood vessels are clearly visible as linear objects with higher intensity: comparison of the vessels before (**a**) and six days after the implantation (**b**) shows that vessels in the lower right corner became the tumor blood supply vessels (much brighter vessels indicating more blood), while the intensity of other vessels remained constant or even decreased.

The mouse was imaged using our custom-made laboratory HSI system before the tumor cells’ implantation and six days after the implantation. From the recorded images, erythema index maps [37] were calculated (Figure 13). The erythema index is a simple metric calculated as a logarithm of the ratio of images taken in the green and red part of the visible spectrum and is commonly used to describe the redness of biological tissues, which is directly related to the amount of blood in the tissue. In Figure 13, blood vessels are clearly visible as linear objects with higher intensity of the erythema index: comparison of the vessels before (Figure 13a) and six days after the implantation (Figure 13b) shows that vessels in the lower right corner became the tumor blood supply vessels (much brighter vessels indicating more blood), while the intensity of other vessels remained constant or even decreased. This example clearly demonstrates the applicability of HSI for monitoring of tumor vasculature evolution.

### 3.10. Example #3 of HSI-System Application in the Biomedical Field: Bruise Imaging

In the third example, our custom-made laboratory HSI system was applied to the imaging of human bruises. The purpose of the hyperspectral imaging was to quantify the concentration of blood and bilirubin, the last being the blood decomposition product and the blood oxygenation in the bruised skin, with the goal to help in estimating bruise age [38].

A bruise of two days’ age was recorded on a knee of a 27-year-old female with Caucasian skin type. The study was approved by the Medical Ethics Committee of the Republic of Slovenia (protocol number 111/02/12). The subject signed an informed consent form and filled out a questionnaire providing information on her age, gender and lifestyle (e.g., smoking, dietary habits).

**Figure 14:**
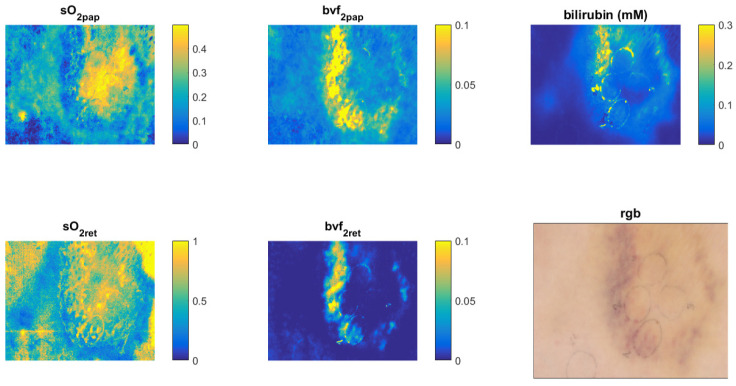
An example of utility of the custom-made laboratory hyperspectral imaging (HSI) system, with shown tissue property maps of a skin bruise extracted from a hyperspectral image using the Inverse Diffuse Approximation Algorithm. The presented tissue parameters are: sO*_2pap_*—blood oxygenation in the papillary dermis, sO*_2ret_*—blood oxygenation in the reticular dermis, bvf*_2pap_*—blood volume fraction in the papillary dermis, bvf*_2ret_*—blood volume fraction in the reticular dermis, bilirubin (mM)–bilirubin concentration in the dermis, and RGB—an RGB image of the bruised skin. All quantities are fractions without units, except bilirubin, which has millimolar units (mM).

The recorded hyperspectral image was analyzed by the Inverse Diffuse Approximation Algorithm [39] to extract the following specific tissue parameters, which were characteristic for bruises: blood oxygenation in the papillary dermis (sO2pap), blood oxygenation in the reticular dermis (sO2ret), blood volume fraction in the papillary dermis (bvf2pap), blood volume fraction in the reticular dermis (bvf2ret) and bilirubin concentration. Distribution maps for each parameter as well as an RGB image of bruised skin are shown in Figure 14. The maps show that skin oxygenation increased in the center and decreased in the boundary regions of the bruise; there was more blood in the boundary region, while the blood concentration in the central region was comparable to that in the normal skin. The bilirubin concentration increased in the boundary region as well. These findings agree well with the visual inspection of the skin (the RGB image), showing that the bruise was most pronounced in the boundary region. The presented quantitative results extracted from the hyperspectral images show that HSI can help doctors to more accurately date a bruise inflicted during, e.g., domestic violence.

## 4. Discussion

In this paper, we have presented the development of a custom-made laboratory HSI system for biomedical applications with a novel LED light source that eliminates the problem of sample heating. Along with the system development, we presented a general framework for calibration, characterization, and testing of any HSI system. Specifically, we proposed the protocol of calibration and characterization of the system, concluding with a verification against a reference technique using liquid ink samples. The main objectives of the study, validation of the system and proposal of validation protocols, were thus achieved. The development of the system was guided by a set of required specifications based on the nature of the biological samples to be imaged. During the characterization and verification of the system, the system performance met and, in multiple requirements, even surpassed the required minimal performance standards set forth before designing the system. During the resolving power tests, a decrease in the resolving power by increasing wavelength was observed for the 50 mm objective lens. Although the decrease needs to be considered, it does not pose obstacles for biomedical imaging applications, in which features in the imaged tissues become typically blurred due to the nature of light–tissue interaction. Furthermore, we have demonstrated the merit of the modular system design by three biomedical applications: (i) multimodal, HSI-3D laser profilometry imaging of a human hand, (ii) monitoring of tumor vasculature evolution in a murine tumor model, and (iii) a skin bruise inspection in a human subject.

A major advantage of our HSI system is the modularity inherent in its design. Our system is well adapted for variable geometries, which is an essential feature for any HSI application in the biomedical field. Using different objective lenses, both the field of view as well as the system resolution can be modified to fit a specific application. The system can also use different light sources, making it easy to expand its use, for example, in fluorescence imaging and Raman spectroscopy. In this way, we have also achieved the secondary objective of the study. Furthermore, the modular system design enables integration of additional imaging modalities such as thermal imaging and 3D laser profilometry, which, in turn, facilitate the acquisition of multi-modal images, offering a depth of insight that is not present in systems employing only spectral imaging. We believe that this modularity and versatility of our system can help in the development of new imaging applications and protocols for those specific samples for which existing commercial systems do not provide sufficient adaptability.

To our knowledge, this is the first custom-made HSI system that uses an LED based light source for reflectance imaging which covers the entire spectral range from 400 nm to 1000 nm. This is especially important for biomedical applications because our illumination system does not heat the samples. Our system can be easily upgraded by also using LEDs as a transmittance source, as well. However, Modir et al. [40] reported in a recent publication that LED can also be used for HSI endoscopic imaging.

The characterization and calibration protocols presented are, although based on a push-broom methodology HSI, in fact generalizable to other systems employing different methodologies, given they are appropriately modified. Such protocols may play a vital role in the development of novel systems and their standardization, which is a prerequisite for the introduction of quantitative imaging metrics and successful implementation of multicentric studies performed on different devices.

An exciting future step is to make our HSI system more compact and offer it to other researchers in the market. HSI in biomedicine is still in its infancy compared to other imaging modalities, and its progress depends on the development of reliable and versatile systems that will be able to answer clinical questions. Despite the early stages of HSI in biomedicine, the many successful preliminary studies being performed around the globe merit future development. Novel technologies, such as the presented LED light source and protocols that pave the way for standardization, are thus of great importance.

In summary, our effort provides both a technological framework and a novel LED light source for spectroscopy which may improve technological standards in HSI system design and expand research opportunities and may be of interest to engineers, physicists, and clinicians.

## Figures and Tables

**Table 1:** Spatial calibration of custom-made laboratory HSI system. Distance on the Ronchi grid *x* that spans *n* pixels for each objective lens gives the size of the pixel ∆*x* on the image plane. Calibration is performed for 2× binning along the spatial direction.

Objective	*x* (mm)	*n* (px)	Δ*x* (mm)
17 mm (1.0 lp/mm)	50	401	0.12
17 mm (0.5 lp/mm)	50	401	0.12
50 mm (1.0 lp/mm)	38	1173	0.03
50 mm (0.5 lp/mm)	39	1205	0.03

**Figure A1:**
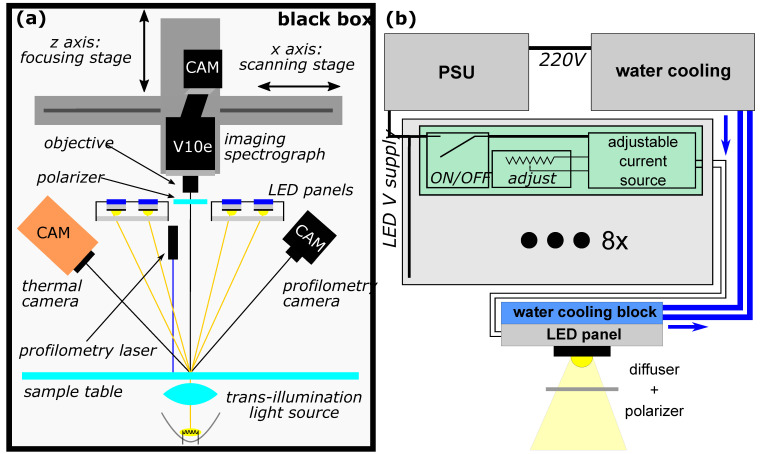
Outline of the custom-made laboratory hyperspectral imaging (HSI) system set-up. (**a**) Outline of the whole system. (**b**) Outline of LED panels with power supply electronics and water-cooling modules. Legend: CAM—camera, V10e—imaging spectrograph, PSU—power supply unit.

**Figure A2:**
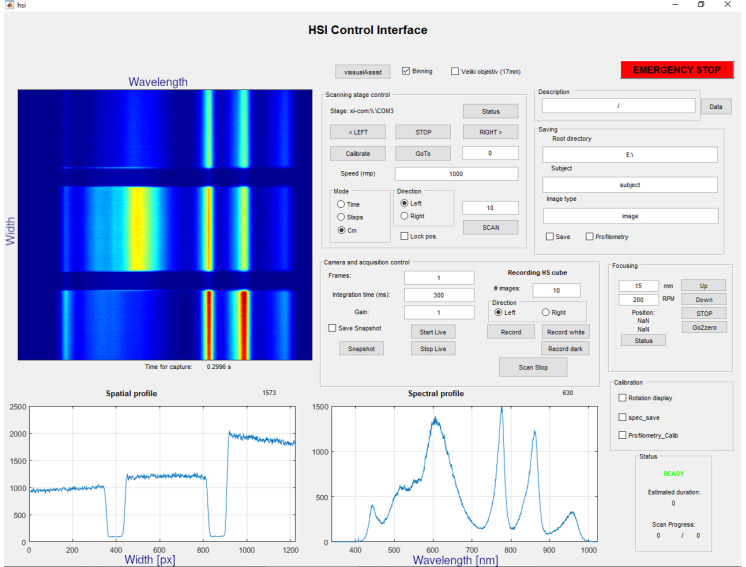
Screenshot of the acquisition software of the custom-made hyperspectral imaging (HSI) system. Control software implemented in MATLAB is showing a color standard (ColorChecker Passport, X-Rite Inc., Grand Rapids, MI, USA). At the bottom, the spectral view and spatial profile along with the scanning line at approximately 500 nm are shown. Additional clarification labels are added to the screenshot in blue.

**Figure A3:**
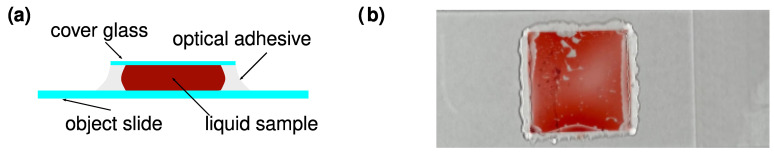
Experimental cells with diluted ink. Ink for verification is enclosed between the object and cover glass and sealed with optical adhesive, as shown in (**a**). A finished cell with red ink is shown in (**b**).

## Data Availability

The data that support the findings of this study are available upon reasonable request from the authors.

## Abbreviations

Throughout the article, the following abbreviations are used:

FOV—field of view
FWHM—full width at half maximum
HNL—helium-neon laser
HSI—hyperspectral imaging
LED—light emitting diode
MP—megapixel
NIR—near infrared
PCB—printed circuit board
PSU—power supply unit
PTFE—polytetrafluoroethylene
RGB—red, green, and blue
USAF—United States Air Force
USB—universal serial bus
UV—ultraviolet
VIS—visible

## Appendix A. Development of the Custom-Made Laboratory HSI System

This section outlines the development of the custom-made laboratory HSI system. The description of the system is broken into multiple categories. Specifically, the following sections describe the imaging part of the system, the light source and the software.

### Appendix A.1. Imaging Components

The core of the system comprises an ImSpector V10e (Specim, Oulu, Finland) imaging spectrograph (slit size 30 µm). The V10e has low spectral smile and keystone distortions, so, typically, no additional corrections are needed. To record the image, a CMOS camera (Blackfly S BFS-U3-51S5M-c, FLIR Integrated Imaging Solutions, Richmond, BC, Canada) with 5 MP resolution (2448 × 2048 pixels), detector size of 2/3 and USB 3.0 connection is used. The detector model is a Sony IMX250 with a pixel size of 3.45 µm. The maximum spectral sensitivity of the camera is at 530 nm (61.82%) and is still 50% at 400 nm and 700 nm; at 1000 nm, it drops to a few percentage points. The camera is aligned so that the larger detector dimension corresponds to the spatial direction of the spectrograph. Since this resolution is rather large for acquisition of objects in the range of 10 cm by 10 cm, where image sizes reach 5 GB and more, the camera usually operates in the binning mode, where spatial pixels are summed in pairs of two, achieving a better signal to noise ratio, and at an effective camera resolution of 1224 × 2048 pixels. Minimal information is lost due to this binning since the actual spatial resolution is below the pixel size in the object plane. To collect light into the spectrograph, two different objective lenses, a 50 mm Xenoplan 2.8/50-0902 and a 17 mm Xenoplan 1.4/17-0903 (Schneider Kreuznach, Bad Kreuznach, Germany), are used. The numerical apertures of the lenses are 0.36 and 0.18 for 17 mm and 50 mm lenses, respectively. Different lenses are needed to adjust FOV and resolution according to the application at hand; small samples (in the range of cm) are imaged with a 50 mm lens, whereas a 17 mm lens is used for larger objects (in the range of 10 cm), thereby sacrificing some spatial resolution for an increase in FOV. An outline of the system set-up is shown in Figure A1.

The imaging spectrograph works as a push-broom system, acquiring only one spatial and spectral dimension at a time. Whole FOV is imaged by scanning in the direction perpendicular to the spectrograph slit. Since moving the sample can be difficult and cumbersome, the imaging head is scanned by means of a motorized translation stage with enhanced loading capacity (8MT195X-340-2.5, Standa, Vilnius, Lithuania) instead. A USB controller (8SMC4-USB-B8-1, Standa, Vilnius, Lithuania) is used to control the stage via a personal computer. For system focusing, a custom-made computerized translation stage for *z*-axis movement is employed.

The CMOS camera has a large dynamic range which must cover a broad range of incoming light intensities. However, the camera has a non-linear response in a part of the range of intensities, therefore, non-linearity correction must be applied to eliminate the non-linearity artifacts in the spectra. The camera non-linearity was measured by measuring reflectance from a reflection standard with almost 100% reflectance for a wide range of camera integration times. The measured non-linearity was implemented in the non-linearity correction algorithm which was used on the measured images. More information about the non-linearity can be found in Dolenec et al. [24].

### Appendix A.2. The Light Source

For reflectance imaging, a custom-made LED-based light source is used [24]. The light source consists of two pairs of two LED panels arranged symmetrically along the recording line. This arrangement, in combination with the natural divergence of the light emitted by LEDs, ensures a uniform illumination of a sample. Each panel features two types of LED diodes, and, when combined, they cover the whole spectral range from 400 nm to 1000 nm. For broadband white light illumination, each panel uses ten white LEDs (LCW H9GP, Oslon Black Warm White, Osram, Munich, Germany); on each of these two panels, white LEDs are interlaced with ten 780 nm LEDs (SMB1N780D, Roithner Lasertechnick, Vienna, Austria). To achieve illumination in the near infrared (NIR) part of the spectrum, the second pair of panels uses a set of ten 850 nm LEDs (SFH 4715S) interlaced with ten 940 nm LEDs (SFH 4725S) each (both Olson Black, Osram, Germany). Thus, in total, 80 high power LEDs are used for sample illumination. Due to the large number of LEDs, the heat dissipated by the panels must be removed from the system enclosure. This is achieved by means of a custom-made closed loop water cooling system. All panels are connected to a water distribution hub, located in the system, via silicone tubing. From the distribution hub, feed and return lines are led into the water cooler, where a large radiator at room temperature is used to cool the water siphoned by a submersion pump via forced air cooling. This setup ensures a constant temperature of the light source and thus minimizes changes in the light spectrum due to the temperature change. The LEDs are powered by an adjustable current source (RCD-24-0.70, Recom Power, Neu-Isenburg, Germany); each channel, i.e., the LED type on one side of the system, can be turned on and off individually. Additionally, to mitigate differences in panel resistance and power supply PCB performance, each channel current—and, in turn, LED brightness—can be adjusted individually by means of a potentiometer on the control box, as depicted in Figure A1.

Imaging with transmitted light is performed by placing a halogen light source (MR16 bulb 12 V 100 W, Osram, Germany) below the glass imaging plate and collimating it using a set of appropriate lenses (16 mm aspheric condenser, ACL2520U, Throlabs, Newton, NJ, USA).

### Appendix A.3. Specular Reflection Minimization

For specular reflection minimization from the surfaces imaged, a pair of linear polarizers with axes of polarization rotated by 90°—a configuration commonly called crossed polarizers—is used. A wire-grid polarizer (Bolder Vision Optik, Boulder, CO, USA) is placed in front of the camera so that the polarization axis could be rotated and adjusted as needed. To polarize the light impinging on the sample, a pair of combined polarizer-diffusers (Bolder Vision Optik, Boulder, CO, USA) attached to a glass plate is placed in front of the LED panels. Diffusers in these polarizers improve homogeneity of illumination of the sample. The location of the combined polarizer-diffusers is shown in Figure A1 as the gray line intersecting the illumination beam.

### Appendix A.4. Additional Components

An optional thermal imaging camera is attached to the imaging head, as depicted in Figure A1. In this way, additional information about the sample surface that could be of interest in inflammation related diseases, such as arthritis, is obtained.

A 3D model of a sample surface is obtained using a custom-made 3D laser profilometer that simultaneously measures the surface of the sample [25,26,27]. The profilometer uses a laser line projector positioned on the vertical axis to project a line on the sample. Due to the sample surface curvature, this line is distorted, and its shape is recorded by means of a secondary camera located off-axis; by triangulation, elevation of individual points along the line is calculated. The 3D model provides the information about sample thicknesses, aids the quantitative analysis [28] and is used to correct intensities in images of curved samples [29]. To prevent the interference between the spectral and profilometric imaging, both acquisition lines are separated by a few mm.

### Appendix A.5. The Software

The system is controlled by a custom-made software developed in the MATLAB environment (R2016b, MathWorks, Natick, MA, USA). The acquisition software supports all the main functionalities of the system, such as programming the scan, controlling the focus distance, previewing the data and saving the metadata equipped measurements. Additionally, the acquisition software offers valuable tools to test the system’s temporal stability by recording a reference spectrum and comparing it to the current spectrum obtained from a live view. A screenshot of the acquisition software is shown in Figure A2.

## Appendix B. Preparation of Sample Cells

To prepare sample cells, a thoroughly cleaned cover glass (Asistent, Germany) was placed on the object microscopy slide (Asistent, Germany) and the edges were then sealed by UV cured optical glue (NOA-63, Norland Products, East Windsor, NJ, USA). The glue was first applied at the corners of the cover slip and left to seep between both glass layers; the cover slip was fixed by curing the glued corners using a UV lamp (365 nm LED). Afterwards, two opposing sides were sealed in a similar fashion to create a channel, into which few µL of pure fountain pen ink (E. Leclerc, Ljubljana, Slovenia) were suction pulled after being pipetted at the edge of the channel; to preserve the standards, the remaining edges were sealed and UV cured. A schematic of a cell is shown in Figure A3. The thickness of the sample cells as measured by the microscope was approximately 100 µm, with deviations across the sample cells of about 20%. As can be seen in Figure A3, the resulting cell samples were not perfectly homogeneous (i.e., concentration of the pigment varied across the sample) due to two factors: (i) variation in the cell thickness and (ii) separation of the pigment in the ink from solvent (water).
